# Exploring digital interaction and positive youth development in a sample of Spanish undergraduates

**DOI:** 10.3934/publichealth.2025052

**Published:** 2025-10-23

**Authors:** Esther López-Bermúdez, Gina Tomé, Diego Gómez-Baya

**Affiliations:** 1 Department of Social, Developmental and Educational Psychology, Universidad de Huelva, Huelva, Spain; 2 School of Health Sciences, Universidade Europeia, Lisboa, Portugal

**Keywords:** internet use, positive youth development, youth well-being, undergraduate, Spain

## Abstract

This study examined the associations between different types of internet use and Positive Youth Development (PYD) among Spanish university students, considering gender differences. A total of 1779 undergraduate students (65.9% men, *M* age = 20.32, *SD* = 1.84) from ten universities in Andalusia (Spain) completed online self-report measures assessing the five dimensions of PYD and some internet use behaviors, including social networking, online gaming, reading, surfing or looking for information, music-related activities, and e-commerce. Descriptive analyses indicated moderate levels of overall PYD, with the highest scores in Caring and Character and the lowest in Competence. Correlation and hierarchical regression analyses showed that the most frequent online activities were social networking, surfing or looking for information, and playing or downloading music. Social networking and online gaming were negatively associated with PYD, whereas reading or looking for information was positively associated. Significant gender differences emerged across all variables, with women reporting more frequent social media use and higher Character and Caring, and men showing more frequent gaming activity and higher Competence and Confidence. The findings underline the distinct associations between specific internet activities and PYD dimensions. The results suggest the importance of promoting constructive digital engagement and mitigating potentially harmful practices. Gender differences should be considered when adopting programs to the needs of each group.

## 1. Introduction

The widespread integration of internet use into daily life, especially among young adults, has intensified interest in its potential implications for psychosocial development. University students frequently engage in both productive online activities, such as educational research and communication, and recreational ones, including social media use, video gaming, and online shopping [1],[2]. The excessive use of the internet and especially social media has been associated with negative consequences in adolescent and youth samples. A recent meta-analysis by Galanis et al. [3] showed the detrimental impact of TikTok use on depression and anxiety. In this line, recent research has concluded that social media is associated with sleepiness and poor sleep among adolescents and young people [4],[5]. Helm et al. [6] observed that high social media use was related to less meaning in life through increasing social isolation in the US. Pedrouzo and Krynski [7] recently recommended safer use of social media in order to prevent its detrimental consequences. Also, some psychological factors, such as psychological distress, low conscientiousness trait, motor impulsivity, and cognitive distortions, have been associated with this problematic internet use [8]. While much of the literature has focused on the negative consequences of problematic internet use (PIU) [9],[10], this study instead addresses the general time spent on various online activities, acknowledging that not all high-frequency usage is inherently maladaptive [9].

To understand the implications of internet use on youth development, Positive Youth Development (PYD) provides a useful framework for understanding how young individuals can thrive during the transition from adolescence to adulthood. This model identifies five dimensions, known as the 5Cs: Competence, Confidence, Connection, Character, and Caring [11],[12]. When these assets are integrated into a life trajectory that fosters contributions to oneself, the family, the community, and society, a sixth C, called Contribution, emerges [13]. Research has shown that these dimensions are positively associated with mental health, well-being, and adaptive developmental outcomes [14],[15]. Overall, PYD promotes a strengths-based approach that enhances resilience and Competence during the transition to adulthood [16].

In parallel with this developmental framework, concerns have arisen regarding the increasing amount of screen time among young adults, particularly in university settings. The Canadian 24-Hour Movement Guidelines for Adults (aged 18–64) recommend limiting sedentary behavior to a maximum of eight hours per day, including no more than three hours of recreational screen time [17]. However, university students typically exceed these thresholds, reporting an average of 7.29 hours of sedentary time per day (self-reported), and up to 9.82 hours when measured with accelerometers, with computer use being the most frequent activity [18]. This prolonged screen exposure has been identified as a potential contributor to the rising prevalence of mental health concerns among youth [19]. Notably, time spent on social media has been specifically associated with body dissatisfaction, particularly among Canadian adolescents [20].

While PYD has traditionally been applied to adolescent populations, recent studies have extended its relevance to emerging adults [21]. For example, research in Croatia and Spain has linked the 5Cs to lower levels of depression among university students, with gender differences indicating higher levels of caring among women and greater Competence among men [15],[22]. In contrast, screen-based recreational activities such as passive social media consumption or excessive gaming have been associated with mental health risks, including stress, anxiety, and loneliness [13],[23]–[28]. However, using the internet for reading or information seeking may positively relate to PYD indicators such as Competence and Character [29].

To date, only the study by Joorabchi and Haghighat [30], with a sample of Malaysian undergraduates, has examined the relationship between PYD and internet use among university students, controlling for degree of study and income level. These authors found mediating effects for internet use gratification on PIU and all PYD dimensions except for Confidence. A related study with adolescents in Spain indicated that weekday internet use was negatively associated with overall PYD, particularly when driven by emotional or compulsive motives [29].

Thus, although some cross-cultural evidence has been collected about internet use and some mental health problems [31], more research is still needed to address the influence on the 5Cs of PYD across countries. In the case of Spain, the use of the internet is generalized among youth, and most of them use social media and instant messaging to communicate with each other (99.9% of women and 99.6% of men). High prevalence of problematic internet use was observed in undergraduate samples, reaching 21% [32]. Some gender differences have been observed in internet use in the Spanish general population, with a gender gap showing a greater use in women [33]. Data with Spanish adolescents indicated that girls reported a greater use of social networks [34] and boys indicated more time and money spent in videogames [35]. However, more research is needed to examine the gender differences in the specific internet uses in Spanish youth.

Given these insights, this study aims to explore the relationships between the types of internet use and the five dimensions of PYD among Andalusian university students in Spain. We hypothesize that: 1) screen-based recreational activities will show negative associations with the 5Cs, while productive internet use will correlate positively; and 2) gender differences will emerge, with women spending more time on social media and men spending more time on gaming.

## 2. Materials and methods

### 2.1. Participants

The participants were undergraduates from 10 universities in the region of Andalusia (Spain): the University of Almería, the University of Cádiz, the University of Córdoba, the University of Granada, the University of Huelva, the University of Jaén, the University of Málaga, the University of Sevilla, the Pablo de Olavide University (Seville), and the Loyola University (Seville and Córdoba). A convenience sampling method was used to ensure heterogeneous geographical distribution. The selection of degree programs was done randomly in each university, and the academic year was also randomly selected. To reach a power (1–*β*) of 95% and a type I error rate (*α*) of 5%, accounting for the potential drop-out rate, a total of 1320 subjects were estimated to be necessary for the sample using G*Power.

### 2.2. Data collection procedure

This study is part of the first data collection occurring from March to June 2023 because of a longitudinal study with a mixed methodology (quantitative and qualitative). For this study, the procedure was quantitative, using an anonymous online self-report measure administered through an online survey via Qualtrics. All universities agreed to participate in the research, and each participant was provided with an information sheet. The exclusion criterion was related to age, excluding participants older than 29 years. The inclusion criterion was enrollment in a degree program at a participating university.

Different professors from each university were contacted to share the online questionnaire with their students. A total of 1728 professors were contacted, and 393 agreed to share the survey with their students, so that the acceptance rate was 22.64%. The students completed the online questionnaire using their computers, which included scales on positive youth development, lifestyle, and sociodemographic variables. This took about 30 minutes during class time. Participation was voluntary, participants were informed about the use of their data, and written online informed consent was provided. Participants did not receive any reward for participating in this study and were allowed to leave the questionnaire at any time. The study was approved by the Bioethics Committee of the University of Huelva on January 10, 2019 (UHU–1259711).

### 2.3. Instrument

#### 2.3.1. Positive youth development

The short version, developed by Geldhof et al. and adapted to Spanish with a sample of Spanish adolescents and youth by Gómez-Baya et al., was administered [36]–[38]. This instrument consists of 34 items distributed in 5 subscales in correspondence with the 5Cs: perceived Competence (6 items related to positive self-efficacy in different areas), Confidence (6 items related to positive self-esteem), Character (8 items, respect for the norms of society and culture), Connection (8 items about positive relationships with others), and Caring (6 items about sympathy and empathy for others). The indicators were assessed following a 5-point Likert scale (from strongly disagree/not at all important/never or almost never to strongly agree/very important/always). The overall score was calculated by averaging the five dimensions of the 5Cs. The questionnaire had notable internal consistency reliability in the overall scale (*α* = 0.85), as well as in the four Cs (Perceived Competence: *α* = 0.69; Confidence: *α* = 0.77; Connection: *α* = 0.74; Caring: *α* = 0.83). The Character showed less internal consistency (*α* = 0.58).

#### 2.3.2. Internet use

In the lifestyle section of the questionnaire, five specific items were included to evaluate the types of activities performed on the internet, validated in prior research with Spanish youth [29]. Specifically, the sample was asked about time spent on social networks, playing online, reading, surfing or looking for information, playing or downloading music, and searching, selling, or buying products. Respondents selected from six options: none, half an hour or less, about 1 h, 2–3 h, 4–5 h, and 6 or more hours. Poor internal consistency was observed in these five items (*α* = 0.52) because these indicators examine very different types of activities. Furthermore, exploratory factor analysis revealed good factorial validity with KMO = 0.682, Bartlett's Test of Sphericity *χ^2^* = 537.90, *p* < 0.001, with one factor with eigenvalue = 1.76.

### 2.4. Statistical analysis

The data were analyzed using SPSS 27.0 for Windows (SPSS Inc. Chicago, IL, U.S.A.). The sample size calculation was performed using the G*Power software (version 3.1.9.7, Universität Kiel, Germany). First, descriptive statistics (i.e., means and standard deviations) were presented for PYD and the frequency distribution of variables related to internet use. Second, differences in overall PYD and the 5Cs based on the kind of internet use were examined using a Student's *t*-test. This test was also used to study gender differences. The effect size was indicated to show the statistically significant differences in the 95% confidence interval [Cohen's *d* (<0.199 negligible; 0.20–0.499 small; 0.50–0.799 medium; ≥0.80 large)] for the Student T test; Cramer's *V* [(<0.4 small; 0.5–0.13 moderate; 0.14–0.22 large) for the *χ^2^* test] [39]. Third, Pearson correlation analysis was performed to determine the associations between the 5Cs of PYD and internet use. Fourth, a hierarchical linear regression analysis was conducted to explain the overall PYD and the 5Cs on sociodemographic variables and different internet use patterns. The Durbin–Watson test (DW) was conducted to detect autocorrelation in the residuals from a regression analysis.

### 2.5. Ethics

The participants provided their written informed consent to participate in this study. The studies involving human participants were reviewed and approved by the bioethics committee of the University of Huelva on 10 January 2019 (UHU–1259711), applying the guidelines of the Declaration of Helsinki.

## 3. Results

### 3.1. Descriptive statistics

The final sample consisted of 1779 university students (age range = 18–29, *M* age = 20.32, *SD* = 1.84, 65.9% were women). The degrees were distributed into Social Sciences and Law (49.4%), Sciences and Engineering (22.2%), Arts and Humanities (15.1%), and Health Sciences (13.3%). In terms of the academic year, 55.1% of the participants were enrolled in the first year, 39.1% in the second year, and 5.7% in the third to sixth year. Most of the students lived in their family homes (47.1%) and only 2.9% shared an apartment with other students. Most of the participants were not seeking employment (64.9%), while 21% were engaged in temporary jobs. Concerning habitat, 37.5% lived in cities with more than 300,000 inhabitants, 32.4% lived in cities of 50,001–300,000 inhabitants, and the rest of the students lived in small towns or in rural areas.

Table 1 presents the descriptive statistics (mean and standard deviation) of the 5Cs and the overall PYD in the total sample, by gender. The results indicated a moderately high overall PYD (*M* = 3.65, *SD* = 0.40) on a scale of 1 to 5. Within the 5Cs, the highest values were observed in Caring (*M* = 4.14, *SD* = 0.66) and Character, while the lowest score was found in Competence. Significant gender differences were observed in several dimensions. Women scored higher in Character and Caring, while men showed higher levels of Competence and Confidence. No significant gender differences were found in Connection and overall PYD.

**Table 1.** Descriptive statistics of 5Cs and overall PYD by gender.

	**Total**			**Women**			**Men**			***t*-tests (Cohen's *d*)**
	
	*M*	*SD*	*IR*	*M*	*SD*	*IR*	*M*	*SD*	*IR*	
1. Character	3.86	0.45	3.12	3.90	0.44	3.12	3.78	0.46	2.75	5.69*** (0.29)
2. Competence	3.00	0.68	4.00	2.87	0.63	4.00	3.25	0.70	3.83	−11.6*** (−0.58)
3. Confidence	3.65	0.68	4.03	3.60	0.69	4.03	3.75	0.65	4.00	−4.57*** (−0.23)
4. Caring	4.14	0.66	3.84	4.28	0.60	3.68	3.87	0.70	3.83	12.9*** (0.65)
5. Connection	3.61	0.60	4.00	3.62	0.60	4.00	3.59	0.60	3.87	1.05 (0.05)
6. PYD	3.65	0.40	3.06	3.65	0.40	3.06	3.65	0.41	2.73	0.26 (0.01)

Note: *M*: Mean; *SD*: standard deviation; *IR*: interquartile range. ***p < 0.001; **p < 0.01; *p < 0.05.

**Figure 1.**
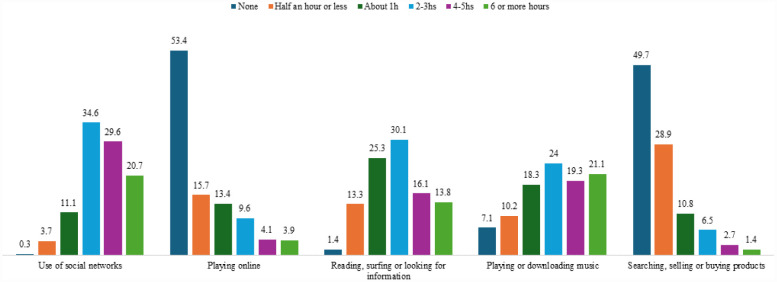
Distribution of time spent on different uses of the internet.

Figure 1 shows that “Playing online” and “Searching, selling or buying products” were activities in which a high percentage of people did not spend time, with 53.4% and 49.7%, respectively. However, “Use of social networks” (34.6%), “Reading, surfing or looking for information” (30.1%), and “Playing or downloading music” (24%) were activities to which they dedicated between 2 and 3 hours.

**Figure 2.**
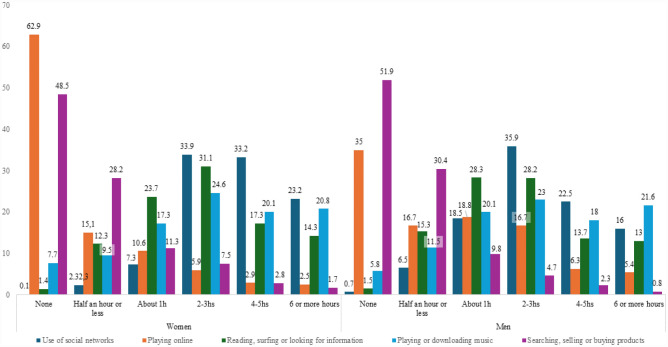
Distribution of time spent on different uses of the internet by gender.

Figure 2 shows the distribution of time spent on different activities by gender. Women reported playing online less, with 62.9% not engaging in this activity compared to 35% of men (*Z* = 11.1, *χ^2^* = 153.90, *p* < 0.001, *V* = 0.30). Moreover, 16.7% of men reported playing online 2–3 hours a day, compared to 5.9% of women (*Z* = 7.3). Some gender differences were observed in the use of social networks, with women reporting a more frequent use (*χ^2^* = 93.68, *p* < 0.001, *V* = 0.23). In total, 33.2% of women used social networks 4–5 hours a day, compared to 22.5% of men (*Z* = 4.7). Moreover, 23.2% of women indicated using social networks for 6 hours or more per day, compared to 16% of men (*Z* = 3.5). Regarding “Searching, selling, or buying products”, “Reading, surfing, or looking for information”, and “Playing or downloading music”, no significant gender differences were detected.

### 3.2. Bivariate correlations between the 5Cs of PYD and internet use

Table 2 presents the bivariate correlations between the 5Cs, overall PYD, and variables related to internet use. The results showed that the “Use of social networks” was not significantly correlated with most of the 5Cs, except for small positive associations with Caring and Connection. Positive associations were found between “Reading, surfing, or looking for information” with Character and Caring. Additionally, “Playing online” showed negative correlations with Character, Caring, and overall PYD. In terms of “Playing or downloading music”, there was a small negative association with Confidence.

**Table 2.** Bivariate Pearson correlations between 5Cs, PYD, and internet use.

	**1**	**2**	**3**	**4**	**5**	**6**	**7**	**8**	**9**	**10**	**11**
**10.** Character	1										
**20.** Competence	0.19***	1									
**30.** Confidence	0.35***	0.56***	1								
**40.** Caring	0.48***	−0.05	0.02	1							
**50.** Connection	0.34***	0.41***	0.47***	0.18***	1						
**60.** PYD	0.66***	0.68***	0.75***	0.48***	0.73***	1					
**70.** Use of social networks	−0.02	−0.02	−0.04	0.06*	0.05*	0.01	1				
**80.** Playing online	−0.11***	−0.01	−0.04	−0.14***	−0.09***	−0.11***	0.09***	1			
**90.** Reading, surfing, or looking for information	0.12***	−0.04	−0.03	0.09***	0.02	0.04	0.21***	0.06*	1		
**100.** Playing or downloading music	−0.02	−0.03	−0.05*	−0.01	−0.03	−0.04	0.26***	0.18***	0.30***	1	
**110.** Searching, selling, or buying products	0.01	0.03	0.01	0.03	0.05	0.03	0.17***	0.11***	0.22***	0.24***	1

Note: PYD: Positive youth development 0. ***p < 0.001; **p < 0.01; *p < 0.05.

### 3.3. Hierarchical regression analysis

Table 3 summarizes the results of six hierarchical regression analyses conducted to explain the overall PYD and the 5Cs based on sociodemographic variables and different patterns of internet use. Standardized coefficients and 95% confidence intervals are presented. Durbin–Watson scores indicated no autocorrelation in the residuals of the regression analyses. Neither age nor academic year significantly predicted overall PYD or any of the 5Cs when internet use variables were included in the models. The variances explained by these models ranged between 2% and 9.6%, suggesting that other factors might play a more substantial role in predicting PYD and its components. The “Use of social networks” positively impacted Competence but had a negative effect on Character. The results pointed out that “Playing online” consistently showed negative effects across all PYD components. “Reading, surfing, or looking for information” was positively associated with overall PYD, Character (*β* = 0.14, *p* < 0.001), and Caring. “Playing or downloading music” had a slight negative impact on overall PYD, and “Searching, selling, or buying products” showed a positive relationship with Connection and Competence. Gender differences were found in several PYD components showing positive effects on Competence (*β* = 0.27, *p* < 0.001) and Confidence, but negative effects on Character and Caring (*β* = −0.28, *p* < 0.001).

**Table 3.** Hierarchical regression analysis of the effects by demographics and variables of Internet use on 5Cs and global PYD.

	**PYD**		**Character**		**Competence**		**Confidence**		**Caring**		**Connection**	
	
	*F* = 4.10*** R^2^ = 0.024 *DW* = 1.95		*F* = 8.16*** R^2^ = 0.046 *DW* = 1.92		*F* = 16.64*** R^2^ = 0.089 *DW* = 1.89		*F* = 3.99*** R^2^ = 0.023 *DW* = 1.94		*F* = 18.05*** R^2^ = 0.096 *DW* = 2.00		*F* = 3.92*** R^2^ = 0.022 *DW* = 2.00	
	
	*B CI*	*β*	*B CI*	*β*	*B CI*	*β*	*B CI*	*β*	*B CI*	*β*	*B CI*	*β*
**Gender**	−0.03, 0.05	0.01	−0.16, −0.07	−0.12***	0.32, 0.45	0.27***	0.10, 0.23	0.12***	−0.45, −0.33	−0.28***	−0.08, 0.04	−0.02
**Nationality**	−0.08, 0.09	0.01	0.04, 0.22	0.07**	−0.12, 0.16	0.01	−0.13, 0.16	0.01	−0.20, 0.06	−0.02	−0.10, 0.05	−0.03
**Age**	−0.04, 0.03	−0.01	−0.02, 0.05	0.02	−0.06, 0.06	0.01	−0.08, 0.04	−0.02	−0.05, 0.06	0.01	−0.07, 0.03	−0.02
**University**	−0.01, 0.01	0.02	−0.01, 0.01	0.01	−0.02, 0.01	−0.01	−0.01, 0.02	0.03	0.01, 0.03	0.06*	−0.02, 0.01	−0.02
**Academic year**	−0.03, 0.04	0.01	−0.05, 0.03	−0.01	−0.05, 0.07	0.01	−0.02, 0.09	0.03	−0.04, 0.07	0.02	−0.09, 0.01	−0.04
**Use of social networks**	−0.01, 0.03	0.03	−0.05, −0.01	−0.06*	0.01, 0.07	0.06*	−0.03, 0.03	−0.01	−0.03, 0.03	0.01	0.01, 0.07	0.07**
**Playing online**	−0.05, −0.03	−0.14***	−0.04, −0.01	−0.08**	−0.08, −0.03	−0.11***	−0.06, −0.01	−0.08**	−0.06, −0.01	−0.07**	−0.07, −0.03	−0.12***
**Reading, surfing, or looking for information**	0.01, 0.03	0.05*	0.03, 0.07	0.14***	−0.04, 0.01	−0.03	−0.04, 0.02	−0.02	0.02, 0.07	0.09***	−0.02, 0.03	0.02
**Playing or downloading music**	−0.03, −0.01	−0.05*	−0.02, 0.01	−0.03	−0.04, 0.01	−0.03	−0.04, 0.01	−0.04	−0.03, 0.01	−0.03	−0.04, 0.01	−0.04
**Searching, selling, or buying products**	−0.01, 0.03	0.05	−0.03, 0.01	−0.02	0.01, 0.07	0.07**	−0.01, 0.05	0.03	−0.02, 0.03	0.01	0.01, 0.06	0.06*

Note. ***p < 0.001; **p < 0.01; *p < 0.05.

The results of the regression analysis by gender (Table 4) revealed some significant associations between internet use and PYD. Table 4 shows the standardized coefficients of the effects included in the regression analyses. For clarity, this table does not include confidence intervals. Durbin–Watson scores also showed no autocorrelation in the residuals of the regression analyses by gender. Gender differences were observed, with a more significant effect to explain PYD in the women sample, where several notable associations emerged. Among the sociodemographic variables, small positive effects were found between nationality and Character, university and Caring, and academic year and Competence. Specifically, “Reading, surfing, or looking for information” had significant positive relationships with PYD, Character, and Caring. “Searching, selling, or buying products” demonstrated a positive relationship with Competence. Conversely, “Use of social networks” was negatively associated with Character and “Playing or downloading music” with Competence. Furthermore, “Playing online” was negatively related to overall PYD, Character, Competence, Caring, and Connection. In the sample of men, demographics did not show any significant effect on PYD or the 5Cs. The “Use of social networks” was positively related to Competence, and “Playing online” was negatively associated with overall PYD, Character, Caring, and Connection. Additionally, “Reading, surfing, or looking for information” was negatively associated with Competence (*β* = −0.16, *p* < 0.001) and positively associated with Character and Caring. There was no observed relationship between Confidence and the studied variables in men.

In summary, the hierarchical regression analyses indicated that although certain types of internet use, particularly reading and information seeking, were positively associated with some dimensions of PYD, others, such as online gaming and excessive social media use, were negatively associated. However, the models explained only a small portion of the variance in PYD outcomes, ranging from 1.3% to 4.3%, and the observed effect sizes were generally small to moderate, suggesting the influence of other unmeasured factors.

**Table 4.** Hierarchical regression analysis of the effects by demographics and variables of Internet use on 5Cs and global PYD by gender.

	**PYD**		**Character**		**Competence**		**Confidence**		**Caring**		**Connection**	
	
	Women *F* = 4.22*** R^2^ = 0.033 *DW* = 2.00	Men *F* = 1.51 *R^2^* = 0.023 *DW* = 1.83	Women *F* = 4.16*** R^2^ = 0.032 *DW* = 1.92	Men *F* = 2.84** R^2^ = 0.043 *DW* = 1.89	Women *F* = 3.98*** R^2^ = 0.031 *DW* = 1.92	Men *F* = 2.73** R^2^ = 0.041 *DW* = 1.85	Women *F* = 2.10* R^2^ = 0.017 *DW* = 1.92	Men *F* = 0.81 *R^2^* = 0.013 *DW* = 1.99	Women *F* = 2.11* R^2^ = 0.017 *DW* = 1.99	Men *F* = 1.88 *R^2^* = 0.029 *DW* = 2.04	Women *F* = 2.95** R^2^ = 0.023 *DW* = 2.05	Men *F* = 2.06* R^2^ = 0.031 *DW* = 1.89
**Nationality**	0.01	0.01	0.06*	0.08	0.01	0.03	0.01	0.01	−0.02	−0.04	−0.02	−0.04
**Age**	0.02	−0.05	0.05	−0.02	−0.02	0.02	0.01	−0.06	0.04	−0.04	−0.01	−0.05
**University**	0.03	0.01	0.01	0.04	0.02	−0.07	0.04	−0.02	0.06*	0.06	−0.02	−0.03
**Academic year**	0.02	−0.02	−0.02	0.01	0.02*	−0.01	0.05	−0.02	0.01	0.04	−0.02	−0.08
**Use of social networks**	0.01	0.07	−0.07*	−0.04	0.01	0.13**	−0.01	0.01	−0.02	0.03	0.06	0.09
**Playing online**	−0.14***	−0.13**	−0.05	−0.12**	−0.14***	−0.07	−0.08**	−0.07	−0.05	−0.10*	−0.13***	−0.10*
**Reading, surfing, or looking for information**	0.09**	−0.02	0.15***	0.14**	0.03	−0.16***	0.01	−0.06	0.10**	0.09*	0.04	−0.02
**Playing or downloading music**	−0.06	−0.03	−0.02	−0.05	−0.07*	0.04	−0.06	0.01	−0.01	−0.06	−0.04	−0.05
**Searching, selling, or buying products**	0.06	0.02	0.01	−0.07	0.09**	0.04	0.05	−0.02	−0.01	0.03	0.06	0.05

Note: Standardized coefficients are described. ***p < 0.001; **p < 0.01; *p < 0.05.

## 4. Discussion

This study examined the associations between PYD, the 5Cs, and various forms of internet use in a sample of Spanish undergraduates, with attention to gender differences. Descriptive results showed moderate overall PYD, with the highest scores in Caring and Character and lowest in Competence, a pattern consistent with prior findings among youth populations [40]. Participants predominantly reported engagement in social media, reading/information browsing, and music-related activities.

Findings supported the first hypothesis: the 5Cs were positively related to productive internet use and negatively associated with recreational screen-based activities. Specifically, social network use showed modest positive associations with Caring and Connection, possibly reflecting its role in fostering empathy, emotional support, and social interaction [41],[42]. However, these positive effects may be offset by potential harms; excessive social media use has been linked to depression, emotional dysregulation, loneliness, peer neglect (phubbing), and body dissatisfaction [43]–[46].

In contrast, online gaming was negatively associated with Caring, Character, and overall PYD. These associations may stem from exposure to antisocial behaviors, cyberbullying, and psychological risks such as anxiety and depression [24],[47]–[49]. Nevertheless, prosocial behaviors could counterbalance such effects and reinforce Caring [50]. Notably, educational digital games in university settings may support Competence by enhancing metacognitive abilities [51],[52].

Regression analyses highlighted the role of nationality, university, and academic year as predictors of PYD and its dimensions, especially among female participants. However, the models explained less than 10% of the variance, suggesting that other contextual and psychological variables may exert stronger influences. However, productive internet use (e.g., reading or researching information) correlated positively with PYD, reinforcing the potential benefits of mindful engagement [29],[30]. In contrast, negative aspects, such as distraction, wasted time, privacy risks, and displacement of health-related behaviors, were noted in prior studies [29],[53]. Furthermore, the literature supports links between problematic digital habits and physical and psychosocial health concerns [54].

Results also confirmed the second hypothesis regarding gender-based usage patterns. Female students reported greater social media use and scored higher on Character and Caring, while male students reported more gaming activity and scored higher in Competence and Confidence, in line with previous research [55],[56]. As suggested by Årdal et al., these gender differences may emerge early in development [57]. Similar trends have been documented in internet usage, with women favoring social interaction and men engaging more in gaming [44],[58],[59]. The prevalence of reading/information browsing in both genders may reflect the academic context of the sample, with women particularly active on social networks [44],[60].

These findings underscore the relevance of promoting critical, reflective, and self-regulated digital engagement. Interventions such as mindfulness training have been found to be effective in reducing internet dependence for emotional regulation [61]. In Spain, the Childhood Platform (Plataforma de Infancia) has promoted responsible, creative, and educational digital use among youth [62], and UNICEF has issued guidelines for safe and respectful social media use [63]. Additionally, the Spanish Network for Health Universities (Red Española de Universidades Saludables, REUS) could serve as a platform for integrating digital wellness into broader health promotion initiatives in higher education [64]. Some psychological variables may moderate the relationship between internet use and PYD. Thus, the promotion of emotional regulation skills, self-esteem, or adaptive coping may increase resilience and foster adaptive technology use [65]. Furthermore, the experience of fear of missing out (FOMO) and neuroticism personality trait may increase vulnerability toward negative internet practices and negative emotional consequences [66],[67]. Social isolation may also confer some increased risk for problematic uses [6]. Furthermore, the promotion of positive internet practices may instead foster positive values and prosocial behaviors [68]. Thus, activities such as participating in collaborative online projects, digital volunteering, or mutual support on social media can be related to some dimensions of the PYD model, such as Caring or Character. Studies suggest that prosocial behavior can act as a protective factor against problematic Internet use [69]. Thus, programs to promote positive internet practices may be designed to aim at increasing PYD in undergraduate samples.

### 4.1. Limitations and strengths

Several limitations should be considered when interpreting these findings. First, the cross-sectional design excludes causal inferences and limits the understanding of the temporal relationships between internet use and PYD. Longitudinal research is needed to clarify the directionality. Second, the study relied on self-reported data, which may be subject to biases such as social desirability or recall errors. Furthermore, the nature of internet use (passive *vs*. active) could not be distinguished, nor could time allocation across daily activities be determined. Although PIU is a relevant construct, it was not evaluated here because it fell outside the study's scope. Incorporating measures of PIU in future research could provide a more comprehensive view. Moreover, unmeasured variables such as personality traits or mental and physical health indicators may mediate the relationship between PYD and internet use. Also, some potential confounders in the impact on well-being can be controlled in future research, such as social isolation. Furthermore, the use of a convenient sample from a single region of Spain limits the generalizability of the results; future research may address these variables by including a sample representative of the entire country.

Despite these limitations, this study employed validated instruments and addressed an important gap in the literature by focusing on PYD outcomes. However, the instrument used to measure internet use showed questionable internal consistency and may limit the robustness of our results. The analysis of gender-based differences and the dual nature of internet use adds depth to our understanding of youth digital behavior in the university context.

## 5. Conclusions

This study contributes to the understanding of the relationship between PYD and internet use in emerging adulthood. Findings suggest that different types of internet activity have distinct associations with the 5Cs, with productive use linked to higher PYD and recreational gaming negatively related to multiple dimensions. Gender differences in internet use and PYD scores were consistent with prior evidence, strengthening the importance of customized interventions.

Given the central role of digital engagement in modern youth life, promoting constructive and intentional online behaviors may enhance psychological well-being and personal growth. Encouraging internet uses that support information-seeking, reflection, and social connection, while mitigating those that contribute to disengagement or distress, represents a promising strategy to foster PYD in university students.

## Use of AI tools declaration

The authors declare they have not used Artificial Intelligence (AI) tools in the creation of this article.
